# Quantifying Treatments as Usual and with Technologies in Neurorehabilitation of Individuals with Spinal Cord Injury

**DOI:** 10.3390/healthcare12181840

**Published:** 2024-09-13

**Authors:** Federica Tamburella, Matteo Lorusso, Mario Merone, Luca Bacco, Marco Molinari, Marco Tramontano, Giorgio Scivoletto, Nevio Luigi Tagliamonte

**Affiliations:** 1 Santa Lucia Foundation IRCCS, 00143 Rome, Italy; m.lorusso@hsantalucia.it (M.L.); m.molinari@hsantalucia.it (M.M.); g.scivoletto@hsantalucia.it (G.S.); n.tagliamonte@unicampus.it (N.L.T.); 2 Department of Life Sciences, Health and Health Professions, University Link Campus of Rome, 00165 Rome, Italy; 3 Research Unit of Computer Systems and Bioinformatics, Department of Engineering, University Campus Bio-Medico of Rome, 00128 Rome, Italy; m.merone@unicampus.it (M.M.); l.bacco@unicampus.it (L.B.); 4 Department of Biomedical and Neuromotor Sciences (DIBINEM), Alma Mater University of Bologna, 40126 Bologna, Italy; marco.tramontano@unibo.it; 5 Unit of Occupational Medicine, IRCCS Azienda Ospedaliero-Universitaria di Bologna, 40126 Bologna, Italy; 6 Research Unit of Advanced Robotics and Human-Centered Technologies, Università Campus Bio-Medico di Roma, 00128 Rome, Italy

**Keywords:** neurorehabilitation technologies, neurorehabilitation treatments, spinal cord injury, clustering of neurorehabilitation treatments

## Abstract

Several technologies have been introduced into neurorehabilitation programs to enhance traditional treatment of individuals with Spinal Cord Injury (SCI). Their effectiveness has been widely investigated, but their adoption has not been properly quantified. The aim of this study is to assess the distribution of conventional (Treatment As Usual—TAU) and technology-aided (Treatment With Technologies—TWT) treatments conveniently grouped based on different therapeutic goals in a selected SCI unit. Data from 104 individuals collected in 29 months were collected in a custom database and categorized according to both the conventional American Impairment Scale classification and a newly developed Multifactor (MF) clustering approach that considers additional sources of information (the lesion level, the level of independence in the activities of daily living, and the hospitalization duration). Results indicated an average technology adoption of about 30%. Moreover, the MF clusters were less overlapped, and the differences in TWT adoption were more pronounced than in AIS-based clustering. MF clustering was capable of grouping individuals based both on neurological features and functional abilities. In particular, individuals with motor complete injuries were grouped together, whereas individuals with sensorimotor incomplete SCI were collected separately based on the lesion level. As regards TWT adoption, we found that in the case of motor complete SCI, TWT for muscle tone control and modulation was mainly selected (about 90% of TWT), while the other types of TWT were seldom adopted. Even for individuals with incomplete SCI, the most frequent rehabilitation goal was muscle tone modulation (about 75% of TWT), regardless of the AIS level, and technologies to improve walking ability (about 12% of TWT) and balance control (about 10% of TWT) were mainly used for individuals with thoracic or lumbar lesions. Analyzing TAU distribution, we found that the highest adoption of muscle tone modulation strategies was reported in the case of individuals with motor complete SCI (about 42% of TAU), that is, in cases when almost no gait training was pursued (about 1% of TAU). In the case of cervical motor incomplete SCI, compared to thoracic and lumbar incomplete SCI, there was a greater focus on muscle tone control and force recruitment in addition to walking training (38% and 14% of TAU, respectively) than on balance training. Overall, the MF clustering provided more insights than the traditional AIS-based classification, highlighting differences in TWT adoption. These findings suggest that a wider overview that considers both neurological and functional characteristics of individuals after SCI based on a multifactor analysis could enhance the personalization of neurorehabilitation strategies.

## 1. Introduction

Spinal Cord Injury (SCI) is a devastating condition with high morbidity and mortality, with an incidence rate of 12 and a prevalence of 263 per 100,000 people [1]. This estimate corresponds to about 21 million cases and a consequent significant burden to healthcare systems and economies through high healthcare costs and lost productivity [2]. Spinal cord syndromes are conventionally classified according to the Asia Impairment Scale (AIS) level, based on the International Standards for Neurological Classification of SCI (ISNCSCI) [3], and a wide range of highly different clinical pictures can be identified. The cervical incomplete lesion is currently the most common injury, followed by incomplete paraplegia and complete paraplegia. Lastly, the cervical complete lesion is the least widespread [4]. The severity of the injury is a key factor in the clinical evaluation of individuals with SCI since it is considered the main predictor of neurological recovery, and it is strictly linked to the final level of independence that can be reached [5].

The care of individuals with SCI is multidisciplinary and takes into account the heterogeneity of clinical pictures, the systems involved, and the presence of several secondary health conditions [6]. The consequent overall managing costs are staggering and pose a significant burden to individuals with SCI, their families, and society [7]. Neurorehabilitation is an essential element of the overall care process of individuals following a SCI [8]. It is mainly based on the compensation of functional loss and on the usage of those functions of the sensorimotor system that are still intact to maximally improve independence and facilitate the reintegration into the family and social and work environments [9]. Therefore, the aim of rehabilitation procedures is the improvement in selected outcomes, by exploiting the plasticity of neuronal centers through functional training [10,11,12], to ultimately promote functional recovery and independence in Activities of Daily Living (ADLs) [13].

Conventional rehabilitation strategies, manually performed by physical therapists (PTs), may include different approaches, which must be tailored for each individual based on the severity and the level of the injury. In fact, individuals with an incomplete sensorimotor injury can perform a much wider range of activities than individuals with a complete SCI. The rehabilitation program may include: (i) joint mobilization, muscle tone modulation, or muscle strength improvement; (ii) training on trunk control and transfers to improve postural control and enhance independence during ADLs; (iii) training for standing recovery, also including exercises for the control of weight shifting for static and dynamic balance or gait rehabilitation strategies [14,15].

In the last decades, due to the technological developments in rehabilitation bioengineering, which leverages continuously evolving fields such as robotics [16], wearable and environmental sensors [17,18,19], virtual reality [20,21], and many others, new devices have been introduced in the clinical setting. Their use enabled the implementation of several technology-aided rehabilitation paradigms [22,23] currently mainly adopted as add-ons to conventional treatment [16,24,25,26,27]. Rehabilitation technologies for individuals with SCI available on the market range from simple tools for tone modulation [28], to platforms for balance rehabilitation during both sitting and standing [22,29], up to advanced devices for the recovery of upper or lower limb functions (such as, but not limited to, overground and treadmill-based exoskeletons, with or without body weight support, and end-effector robots). Since the literature recognizes that technological innovation has the potential to enhance rehabilitation paths [30], several studies were aimed at: (i) evaluating the effectiveness of these technologies on specific aspects of patient performance, for example, muscle force, balance, independence in ADLs, etc., often in comparison with conventional rehabilitation [26,31,32]; (ii) identifying possible barriers to rehabilitation technology adoption, by analyzing the perspectives of people with disabilities and of health service providers [33,34,35,36].

Nonetheless, the quantification of the daily actual adoption into clinical settings of SCI rehabilitation [26,31,32] is missing [37]. To fill this gap, we addressed two aspects that have not been investigated in the current literature: (i) the quantification of the overall adoption of technologies in clinical daily practice, compared to conventional treatment, and (ii) the analysis of the specific technological resources adopted in homogeneous groups of individuals with SCI characterized by different neurological and functional features.

Therefore, the aim of this study is to quantify the distribution of conventional treatments (Treatment As Usual—TAU) and technology-aided treatments (Treatment With Technologies—TWTs) and assess the usage of specific categories of technological resources in the neurorehabilitation program of a cohort of individuals with SCI arranged in groups with similar characteristics. The main hypothesis behind this study is that the adoption of TAU and TWT can change significantly among different groups of individuals with SCI, especially in terms of use of technologies (type/aim and amount of usage), because of the vast diversity in the functional abilities following a SCI. Thus, we hypothesize that individuals with different neurological and functional characteristics may adopt differently TAU and TWT, especially in balance and gait rehabilitation.

To this aim, TAU and TWT data were collected, and individuals under analysis were grouped according to the conventional AIS level, based on the ISNCSCI [3], and a new specifically developed MultiFactor (MF) clustering approach that considers the lesion level, the level of independence in the ADLs (before and after the rehabilitation path), and the hospitalization duration, besides the AIS level at admission and discharge. In our hypothesis, the potential differences in TAU or TWT adoption were expected to be more pronounced in the MF clustering, which accounts for a broader range of variables, thus providing a better understanding of rehabilitation needs.

Indeed, we implemented the MF clustering approach to include more variables within the analysis of TAU and TWT adoption since the AIS-based classification does not fully capture the functional heterogeneity in individuals with similar neurological impairments due to SCI. For instance, two individuals classified with the same AIS level may have significantly different functional capabilities: in the case of a cervical injury, the subjects may require extensive assistance for daily activities, while an individual with a thoracic SCI might be more independent. Similarly, the same AIS classification (for instance, AIS C) can be attributed to an individual with a strength score of 1 out of 5 in only one muscle below the SCI, or to an individual only with a preserved anal sphincter contraction, or to another one with strength scores equal to 2 out of 5 in all muscles below the lesion. These differences, not captured by AIS alone, may critically affect the choice of rehabilitation strategies and highlight the need for a more comprehensive classification.

The motivation for this dual analysis lies in the hypothesis that simply clustering individuals according to the conventional AIS-based classification can be ineffective since individuals with SCI, apparently comparable when classified with the same AIS level, can actually pursue very different ranges of neurological and functional recovery, especially after incomplete lesions [38]. Within a single AIS neurological level, individuals with cervical, thoracic, or lumbar SCI may be potentially included. For instance, in the case of cervical SCI and subsequent impairment of upper and lower limbs, the individuals present markedly different neurological features as well as levels of independence in the ADLs than an individual with thoracic injury and lower limb impairment only. Consequently, these aspects will obviously influence the individual’s management, the prognosis, the rehabilitation goals, and the hospitalization duration, and, in turn, technology adoption may vary significantly even among individuals classified with the same AIS neurological level. It is then desirable to make available a clustering method able to group the SCI population into homogeneous subgroups, considering not only the neurological aspect but also the functional one by addressing the level of independence in the ADLs reached within a defined time frame. This clustering is expected to be advantageous for interpreting data about the adoption of different rehabilitation strategies in cases of groups that would otherwise be highly heterogeneous.

We hence expect that individuals belonging to the same AIS category can be too heterogeneous for investigating the specific TAU/TWT distribution and that, contrarily, a MF clustering, accounting for additional variables not considered in the traditional AIS neurological classification, can provide more insightful results on technology use and on its relationship with individuals’ overall rehabilitation program and outcome.

For this study, we have focused on a specific clinical setting, namely, the Spinal Cord Unit within the Neurorehabilitation 1 Department of Fondazione Santa Lucia (FSL) IRCCS in Rome, where rehabilitation technological devices have been progressively introduced along the years in the daily treatments of individuals with SCI. Data have been collected and digitized by means of a newly developed database accessed by a dedicated local Web App.

## 2. Materials and Methods

### 2.1. Study Design

All procedures contributing to this study comply with the ethical standards of the relevant national and institutional guidelines on human experimentation and with the World Medical Association Declaration of Helsinki. Informed consent was obtained from all participants involved in this study. Medical records of all the individuals with SCI admitted to the Spinal Cord Unit of FSL during a period of 29 months were retrieved.

### 2.2. Context

This study was conducted at the Spinal Cord Unit of FSL, where neurorehabilitation, including TAU and/or TWT, was carried out by PTs based on medical prescription following a clinical and functionally devoted evaluation. Treatment prescriptions were patient-specific, and TWT is always adopted in addition to TAU, at least three times per week in sessions lasting approximately 40 min. Periodic team meetings with medical doctors and PTs were held at least every 15 days to assess whether the TWT has to be further adopted or discarded for the remaining rehabilitation program. Each participant performed at least one daily individual rehabilitation session. Based on their clinical condition, individuals may potentially have received additional TAU or TWT sessions. In addition to sensorimotor rehabilitation, individuals also regularly received medical and nursing care, respiratory rehabilitation, occupational therapy, etc. Additionally, a devoted clinical engineering department constantly took care of the maintenance of all the technological devices to guarantee they were continuously and fully working and mitigate as much as possible any possible deterioration and disuse.

### 2.3. Individuals with SCI

Demographic and clinical characteristics recorded at admission in the Spinal Cord Unit were collected (age, gender, SCI etiology, time since injury, AIS neurological level, and functional capabilities in the ADLs based on the Spinal Cord Independence Measure III (SCIM) [39,40]. The total duration of hospitalization (days), the AIS level, and the SCIM score recorded at the discharge were also collected, as well as the treatment goals of each rehabilitation session performed during hospitalization, either based on TAU or TWT, as detailed in Section 2.4.

### 2.4. Treatment as Usual and Treatment with Technologies Categories

The TAU and TWT were conveniently grouped into 4 categories. This classification does not claim to be general or exhaustive, but it is only adopted in the context and for the aims of this study. Indeed, alternative existing technology-aided rehabilitation approaches that are not part of daily clinical practice at the Spinal Cord Unit of FSL were not considered for classification in this study.

The care of an individual with SCI involves setting specific rehabilitation goals, including the PT request for a progression of exercises that allows individuals to gradually improve functional performance [14]. Therefore, individuals with SCI are initially trained to perform all the transfers that are compatible with their capabilities to improve the independence in ADLs. To this aim, a key element is the improvement in trunk control and balance. The next step involves exercises for achieving and maintaining the standing position. As soon as the individual can better manage this functional request, specific study on walking is pursued. Throughout the hospitalization period, individuals with SCI are involved with exercises aimed at the maintenance of joint range of motion and the modulation of muscle tone to reduce spasticity or improve force control. Based on this conventional progression of exercises proposed to gradually improve functional performance, we have grouped the TAU approaches according to the treatment goal as follows: (i) TAU-1: joint range of motion maintenance, muscle tone modulation, and force control; (ii) TAU-2: transfers, balance, and trunk control; (iii) TAU-3: standing position reaching and control; and (iv) TAU-4: gait ability.

Also, the available technologies in the Spinal Cord Unit (Table 1) were conveniently grouped into other 4 categories. However, it was not possible to create equivalent TWT categories that perfectly matched the TAU ones, since not all the treatments proposed by a PT in traditional rehabilitation can be reproduced also with the available technologies. On the basis of their rehabilitation potential and following a criterion of functional performances and upper or lower limb devices, technologies have been grouped as follows: (i) TWT-1: muscle tone modulation, including Cycloergometer (Ce) and Ce aided by Functional Electrical Stimulation (FES Ce); (ii) TWT-2: trunk control, including Balance Plate (BP) and Balance Perturbation Plate (BPP); (iii) TWT-3: gait ability, including Tibialis Anterior FES (TA FES), Lower Limb Overground Exoskeleton (OG Exo), and Lower limb Treadmill-Based Exoskeleton (TB Exo); (iv) TWT-4: upper limb functionality, including Upper limb Game-based Motor Trainer (MT) and Upper limb Motor Imagery Brain Computer Interface (MI BCI).

Specifically the available technologies were: (i) Ce (Motomed, Chinesport, Italy [41,42]), to be used for upper or lower limb muscle tone modulation training during active or assisted limb motion; (ii) FES Ce (RehaMove, Hasomed, Germany [43]), consisting of the aforementioned Ce used in combination with FES stimulating different selected muscles; (iii) BP (Tymo, Tyromotion, Austria [44]), providing posturography-like feedback and gaming to train sitting and standing postural control; (iv) BPP (Balance SD, Biodex, US [45,46]), a BP that includes an actuated tilting system able to provide under-feet perturbations to balance, with different individually adjustable levels of challenge; (v) TA FES (WalkAide, Axio Bionics, US [47,48]), a wearable FES device to be used on the ankle flexor muscles to stimulate and support dorsi-flexion during walking rehabilitation; (vi) OG Exo (Ekso, Ekso Bionics, US [22,23,49]) supporting hip and knee flexion/extension during locomotion with different levels of assistance; (vii) TB Exo (Lokomat, Hocoma, Switzerland [22,50,51]), a gait trainer, including a wearable active orthosis assisting hip and knee flexion/extension and a body weight support with dynamic unloading capabilities; (viii) MT (Pablo, Tyromotion, Austria [52]), a hand-held tool with orientation sensors (to monitor motion) and force sensor (to monitor grasping), providing feedback and including gaming to train upper limb movements; (ix) MI BCI (Promotoer, Italy [53,54,55]), a brain-computer interface, including the visualization of a virtual hand to train the patient in opening/closing movements based on the principle of kinesthetic motor imagery. All the above-reported technological devices were constantly under monitoring and maintenance by the hospital clinical engineering department, to be continuously and fully working.

It is useful to highlight that the team of PTs included 16 professionals with at least 3 years of experience in neurorehabilitation. Additionally, 70% of them have been working at the FSL Spinal Cord Unit for about 12 years. The whole team was fully trained in using almost all the technologies. Indeed, within the first week of enrollment, each member of the team received a specific training from the PT responsible for internal education, and also continuous training updates are guaranteed by FSL periodically. The only exceptions were the TA FES, the TB exo, the OG exo, and the MI BCI, for which not all the PTs were trained. Nonetheless, about 30% of the team was able to use the TA-FES and OG exo devices, whereas about 80% was able to use the TB exo. Moreover, a dedicated hospital service, including a single specialized PT, ensured the provision of TWT based on MI BCI. It is worth noting that this organization did not affect the adoption of TWT that was successfully delivered to all the requiring patients. The number of trained physical therapists was always sufficient to cover patients’ treatment needs, and, in specific cases, dedicated changes in patient–therapists assignments were made to assure that trained PT could be properly assigned as needed.

### 2.5. Data Collection and Storage

For data collection, a custom Web App was developed by using the Atom editor v.1.63 and the Wamp software v.3.3 platform, which includes the web server Apache and the database management system MySQL. The application was developed through the languages of scripting Hypertext Preprocessor (PHP) and HyperText Markup Language (HTML). The implemented Web App allowed the data extracted from medical records to be stored directly in digital format. Specifically, the platform was able to collect and manage the following individuals’ information gathered from the medical records regularly compiled by the members of the clinical team in charge of individuals with SCI: gender, age, AIS level and SCIM score at admission and discharge, hospitalization duration, and specific data on the TAU and/or TWT (number of sessions and treatment goal). TAU and TWT data were collected starting from the first admission day. Indeed, according to medical prescription, TAU administration generally begins as soon as the individual is admitted to the Spinal Cord Unit, while TWT beginning takes into account also expected tolerance to the use of the proposed TWT and the specific contraindications of each device. In addition, the application was used to process and extract data through dedicated queries, thus getting information on selected classes of individuals (e.g., based on neurological lesion level), on the treatment goals of the delivered therapies, or on the number of TWT and TAU sessions delivered in the hospitalization period. The application was designed to be user-friendly and to minimize errors: it allows inserting user inputs only through mouse clicks, and data entry is performed through drop-down menus.

The data collected through the application were stored in a relational database, specifically designed to optimize the management of the information on rehabilitation sessions. The database includes:

- The patient code entity is associated with the admission entity, which contains the information regarding the admission in the Spinal Cord Unit. Each admission can be linked to several rehabilitation sessions (described in the session entity), which in turn can consist of multiple therapies (reported in the therapy entity).
- The user entity, which contains the demographic information of the users who inserted data, as well as the username and password credentials for login operations, and the information related to the role they play within the department. Since the user is allowed to register several patients, she/he can associate the admissions entities with the patient code entities. Furthermore, to optimize the information extraction about therapies performed by individuals with SCI with the same level of injury and AIS, with or without the aid of technological devices, we separately included pathology, AIS, and etiology entities linked to the patient entities.

Within this framework, stringent measures were taken to ensure privacy protection. All data were anonymized, meaning that any information that could potentially lead to the identity of individuals was removed. Additionally, the database was local and not made publicly accessible. Access was restricted exclusively to the researchers involved in this study, further limiting the potential for privacy breaches.

#### Clustering and Data Analysis

For the purpose of this study, we decided to cluster the data according to two different approaches and to analyze the differences in these two possible ways of grouping individuals: (i) AIS-based clustering was adopted as a fairly straightforward and widely recognized method of classifying individuals, that is, considering their AIS score, and (ii) a MF clustering method was introduced to group the individuals by considering the similarity of the groups based on multiple characteristics.

Besides the AIS-based classification, the MF clustering approach was chosen because it integrates additional factors beyond the neurological impairment, such as the lesion level, the level of independence in the ADLs (before and after the rehabilitation path), and the hospitalization duration. This multifactor approach may outline a more holistic patient’s profile, allowing for a deeper analysis of the adoption of TAU or TWT.

To pursue MF clustering, an array representation of each individual was derived by extracting categorical and quantitative groups of features. For the former, SCI lesion level (cervical, thoracic, and lumbar) and AIS score at admission and discharge were considered. For the latter, SCIM scores at admission and discharge and hospitalization duration were considered. Once the individuals’ representations were obtained, each feature was standardized as $X_{i}^{\prime }=(X_{i}-\mu_{i}$)/$\sigma_{i}$, where $X_{i}$ and $X_{i}^{\prime }$ indicate the i-th feature vector among all the samples in the set, before and after the standardization process, respectively, and $\mu_{i}$ and $\sigma_{i}$ are the mean and the standard deviation values. This pre-processing step allows the algorithm to be applied considering the computation of a defined distance metric without impact from different feature magnitudes.

A hierarchical clustering in an agglomerative way with a bottom-up approach was used. The algorithm starts by considering each of the data samples (i.e., individuals) as a single cluster, and then it iteratively merges pairs of clusters. At each elaboration step, the merged pairs are those that minimize an objective function based on a certain definition of distance. The Ward’s method [56] was used as the linkage criterion to minimize the intra-cluster variance in terms of the Euclidean distance. The iterations stop when the algorithm returns a single all-inclusive cluster.

For both AIS-based and MF clustering, the silhouette score [57] was calculated for all the samples. By considering a sample $c_{n}$ belonging to the i-th cluster $C_{i}$, we first computed the intra-cluster distance $a\left(c_{n}\right)$ as the average Euclidean distance $d\left(c_{n},\;c_{m}\right)$ from all other points $c_{m}$ within the same cluster, as follows:

(1) $$a\left(c_{n}\right)=\frac{1}{\left|C_{i}\right|-1}\;\sum_{\forall c_{m}∊C_{i},\;n\neq m}d(c_{n},\;c_{m})$$

and the mean nearest-cluster distance $b\left(c_{n}\right)$ as the average Euclidean distance from $c_{n}$ to all points $c_{k}$ in the nearest cluster that is not a part of, as follows:

(2) $$b\left(c_{n}\right)={min}_{i\neq j\;}\left\{\;\frac{1}{\left|C_{j}\right|}\;\sum_{\forall c_{k}∊C_{j}}d\left(c_{n},\;c_{k}\right)\;\right\}$$

In (2) and (3), ${|C}_{i}|$ and ${|C}_{j}|$ are the dimensionalities (i.e., the number of samples) in clusters $C_{i}$ and $C_{j}$, respectively.

Finally, we computed the silhouette scores $S\left(c_{n}\right)$ and averaged them across all the samples in the dataset as follows:

(3) $$silh=\frac{1}{N}\sum_{\forall n}S\left(c_{n}\right)=\frac{b\left(c_{n}\right)-a\left(c_{n}\right)}{max\{a\left(c_{n}\right),\;b\left(c_{n}\right)\}}$$

where *N* is the total number of samples in the dataset.

By definition, a silhouette score of 1 indicates the best possible separation between clusters. Whereas, negative values mean that a sample might be better associated with another cluster, that is, indicate a closer similarity to that cluster than the one it is currently assigned to. Scores close to 0 suggest overlapping among clusters.

Additionally, a MANOVA statistical test on both clustering methodologies was conducted, investigating Wilk’s Lambda, to test overall differences in TWT adoption with no specific categorization of technologies (i.e., not based on rehabilitation goals).

Further investigation of the differences in the adoption of TWT-1, TWT-2, TWT-3, and TWT-4 and of TAU-1, TAU-2, TAU-3, and TAU-4, across AIS-based clusters and across MF clusters, was conducted by means of the Anderson–Darling test [58] to verify the normal data distribution, followed by the Kruskal–Wallis test [59] to assess whether the observed groups originated from populations with identical medians. This test, a nonparametric alternative to the ANOVA, is suitable to account for multiple groups and does not need equal sample sizes.

## 3. Results

A total cohort of 106 individuals with SCI, 89 males and 17 females, aged 51.32 ± 19.92 years, with traumatic etiology in 60% of cases, was included in this study. The mean time elapsed between the SCI event and admission at the Spinal Cord Unit was 96.22 ± 52.90 days. Data from 104 individuals were considered for the analysis because 2 individuals died during hospitalization.

### 3.1. AIS-Based Clustering

#### 3.1.1. Data from Individuals with SCI for AIS-Based Clustering

**Figure 1:**
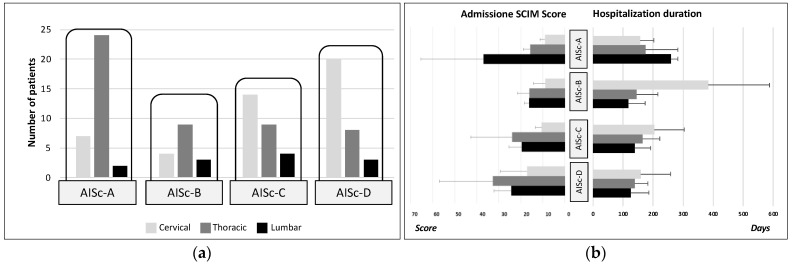
Grouping of the cohort of individuals with SCI according to the AIS-based clustering: (**a**) Number of individuals for each cluster (AISc-A, AISc-B, AISc-C, or AISc-D) grouped according to the lesion level and (**b**) SCIM score at the admission and hospitalization duration. Error bars in the bar graphs indicate the standard deviation.

Four AIS-based clusters were created according to AIS level, that is, including, respectively, individuals with SCI classified as AIS A, B, C, and D levels (AISc-A, AISc-B, AISc-C, AISc-D). AIS-based clustering revealed that most individuals belonged to AISc-A (*n* = 33) or AISc-D (*n* = 30), with fewer individuals grouped into AISc-C (*n* = 27) or AISc-B (*n* = 16). The most represented lesion level was thoracic for AISc-A and AISc-B individuals, whereas lumbar lesion was the least frequent in all categories (Figure 1a). The mean SCIM score recorded at admission was 17.80 ± 12.37. AISc-A and AISc-B individuals had a lower score than individuals with incomplete motor lesions, with the only exception of AISc-A individuals with lumbar lesions. Those with the highest SCIM score were AISc-D individuals. Moreover, individuals with cervical lesions had the lowest SCIM values among all AIS-based categories (Figure 1b).

The mean hospitalization duration was 172.37 ± 101.25 days. Among individuals with incomplete injury (AISc-B, AISc-C, or AISc-D), those with cervical lesions had the longest hospital stay, whereas the earliest discharged individuals were those with lumbar SCI. This pattern was completely reversed in the case of individuals from AISc-A, for whom hospital stays in cases of lumbar lesions were longer than in cases of cervical injuries (Figure 1b).

#### 3.1.2. TAU and TWT Adoption for AIS-Based Clustering

**Figure 2:**
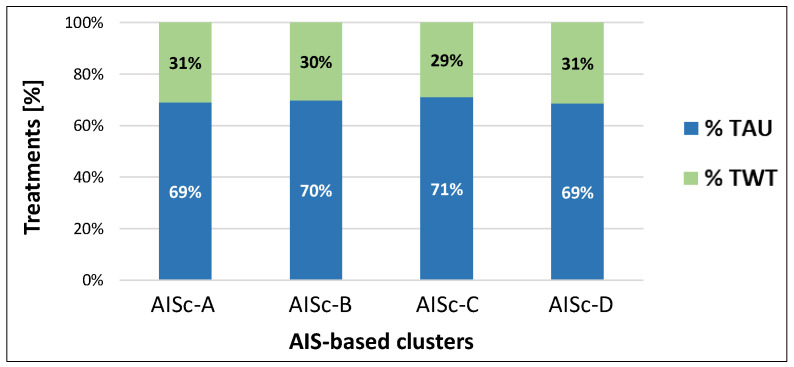
Percentage of TAU or TWT adoption on the total amount of treatments administered for the individuals with SCI classified according to the AIS-based clustering.

TWT represented about 30% of the total treatments received by the sample. No substantial differences were reported among AIS-based categories, ranging from 29% for individuals grouped into AISc-C to 31% for AISc-A and AISc-D clusters (Figure 2).

**Figure 3:**
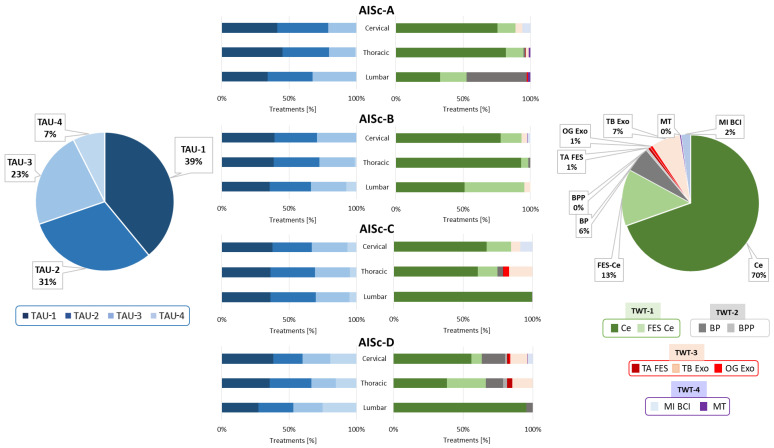
Distribution of TAU-1, TAU-2, TAU-3, and TAU-4 and TWT-1, TWT-2, TWT-3, and TWT-4 for the total cohort of individuals with SCI (pie charts) and for each AIS-based clustering, classified according to the lesion level (cervical, thoracic, or lumbar lesion).

Looking in detail at the conventional treatments across AIS-based categories, TAU-1 was the most adopted one, followed by TAU-2, TAU-3, and TAU-4, respectively (Figure 3).

Notably, TAU-1 is less adopted as the lesion level decreases. Regardless of the level of injury for AISc-A and AISc-B, TAU-4 is almost never present, while its adoption increases as the severity of the injury decreases for individuals with motor incomplete SCI. In particular, TAU-4 was chosen at the expense of TAU-3 for AISc-D.

Considering the analysis of TWT data, TWT-1 adoption was the highest, especially for the Ce (70%), followed by TWT-3, in particular, the OG Exo device (7%), and TWT-2. Finally, TWT-4 devices were the least used (2%). A clear prevalence of a specific device was not found for TWT-2 and TWT-4. Ce (70%) was more commonly used than Ce FES (13%) among all AIS-based categories, regardless of the level of impairment. In cases of complete motor lesions, TWT-2 devices were exclusively used for AISc-A individuals, in particular for those with lumbar SCI. For both AISc-A and AISc-B clusters, different TWT-3 and TWT-4 devices were used, but specifically, the OG Exo and MT were used for individuals with the highest level of impairment only. In cases of AISc-C and AISc-D, individuals received TWT-3 and TWT-4. The only exception was the exclusive use of the TA FES for individuals from AISc-D with cervical and thoracic SCI.

### 3.2. Multifactor Clustering

**Figure 4:**
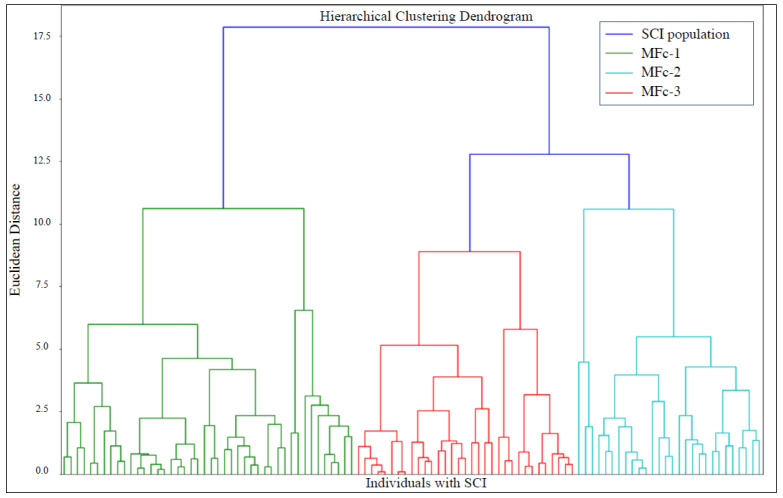
Dendrogram obtained from the hierarchical clustering approach visually representing the arrangement of MF clusters produced by the analysis. Each cluster is represented by a specific color (blue for the whole SCI population, green for MFc-1, light blue for MFc-2, and red for MFc-3).

The MF clustering based on the method described in Section 2.5 produced the dendrogram reported in Figure 4, a tree-like representation of the cluster hierarchy. The Euclidean distance on the dendrogram represents the dissimilarity among clusters, with larger distances indicating more heterogeneity.

Three MF clusters (MFc-1, MFc-2, and MFc-3) were extracted from the dendrogram considering the most relevant distinction among main groups of individuals with SCI. We considered intercluster variance among subclusters at a level below the selected three too small for a further useful distinction. By decreasing the Euclidean distance threshold in the dendrogram, more clusters can be found that show even greater overlap. Hence, individuals belonging to the final three clusters were considered to have homogeneous features.

### 3.3. Statistical Analysis for AIS-Based and MF Clustering Approaches

The silhouette score [57] for all the samples of the MF clusters was equal to 0.02, whereas AIS-based classification resulted in a worse silhouette value of −0.11 (the negative sign indicates closer similarity among clusters).

For the MF clustering, the MANOVA test resulted in a *p*-value equal to 0.0013, indicating strong statistical significance, whereas for the AIS-based clustering, the *p*-value was equal to 0.0186. These results suggest that, even if both clustering methodologies revealed significant differences among groups of individuals, the MF clustering can better separate clusters, as evidenced by the lower *p*-value. This difference points out the MF clustering ability to capture a broader range of variables that influence the rehabilitation process, beyond the mere severity of the injury.

For both clustering methods, the results from the Kruskal–Wallis test showed statistically significant differences when comparing the adoption of TWT-1, TWT-2, TWT-3, or TWT-4 across the four AIS-based clusters and across the three MF clusters with each other (*p* < 0.01). Only two exceptions were found: (i) for the AIS-based clustering, the median adoption of TWT-4 remained consistent across all clusters (*p* > 0.05); (ii) for the MF clustering approach, TWT-2 displayed a *p*-value of 0.056, marginally above the standard cutoff, suggesting a slight deviation from median uniformity. Specifically, for AIS-based clustering, statistical analysis revealed *p* > 0.05 for the AISc-B and AISc-C clusters in the TWT-4 vs. TWT-2/TWT-3 comparison, and for the AISc-D cluster in the TWT-2 vs. TWT-3 comparison. Conversely, for the MF clustering, *p* > 0.05 was reported only in the MFc-3 in the comparisons TWT-2 vs. TWT-3/TWT-4 and TWT-3 vs. TWT-4.

The results of the Kruskal–Wallis test for the analysis of TAU indicate statistically significant differences when comparing TAU-1, TAU-2, TAU-3, and TAU-4 across the four AIS-based clusters and across the three MF clusters (*p* < 0.01). As for the TWT, an exception was found also in this case, but it was present for both clustering approaches. The median value of TAU-3 remained consistent across all clusters, with a *p*-value of 0.051.

#### 3.3.1. Data from Individuals with SCI for Multifactor Clustering

**Figure 5:**
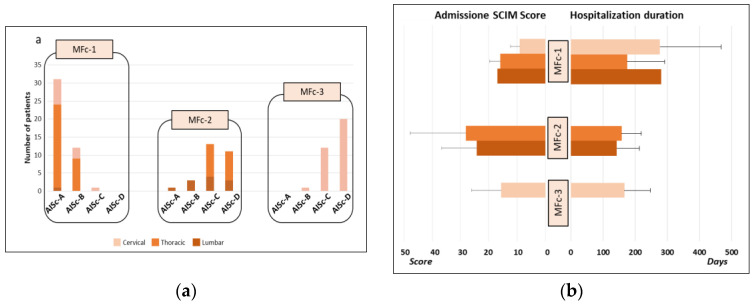
Grouping of the cohort of individuals with SCI according to the MF clustering: (**a**) Number of individuals for each cluster (MFc-1, MFc-2, and MFc-3) grouped according to the lesion level and AIS-based classification (AIS-A, AIS-B, AIS-C, and AIS-D) and (**b**) SCIM score at the admission and hospitalization duration.

MFc-1 was mainly composed of individuals with cervical or thoracic motor complete SCI (it contained no individuals classified as AISc-D and almost no AISc-C individuals). MFc-2 included individuals with a thoracic or lumbar lesion, mainly with motor-incomplete SCI. MFc-3 consisted exclusively of individuals with incomplete cervical lesions and almost no AISc-A individuals (Figure 5a). According to the MF analysis, data showed that the individuals in MFc-1 had the lowest SCIM score on admission and the higher duration of hospitalization. On the contrary, individuals belonging to MFc-2 had the highest SCIM score on admission and the shortest hospitalization (Figure 5b).

#### 3.3.2. TAU and TWT Adoption for Multifactor Clustering

**Figure 6:**
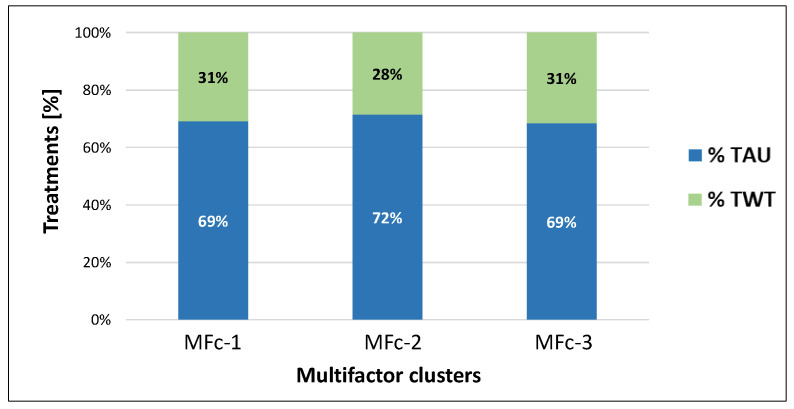
Percentage of TAU or TWT adoption on the total amount of treatments administered for the individuals with SCI, classified according to the MF clustering.

The three MF clusters presented a homogeneous distribution between TAU and TWT (Figure 6), with a minor difference for cluster MFc-2 that received less TWT (28%) than the other clusters (31%).

Overall, considering the conventional rehabilitation, TAU-1 was the most adopted, followed by TAU-2, TAU-3, and TAU-4. Cluster MFc-1 presented the highest TAU-1 and the lowest TAU-4 adoption. Instead, for the other two clusters, TAU-4 covered more than 10% of the total TAU. The data on the TAU-3 were similar among the clusters, while TAU-2 was mainly proposed in cluster MFc-1. For these data, no substantial differences were noted when considering the lesion levels.

**Figure 7:**
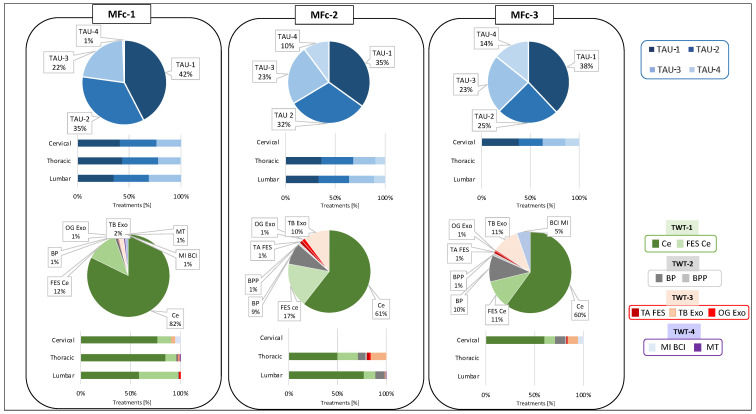
Distribution of TAU-1, TAU-2, TAU-3, and TAU-4 and TWT-1, TWT-2, TWT-3, and TWT-4 for each MF cluster MFc-1, MFc-2, and MFc-3 (pie charts), classified according to the lesion level (bar graphs).

The data on TWT showed that for each of the three clusters, TWT-1 was the most adopted, whereas TWT-4 was the least used. TWT distribution between MFc-2 and MFc-3 was similar and different compared to MFc-1, with the only exception of the MI BCI device that was absent in MFc-2. Compared to MFc-1, MFc-2 and MFc-3 presented a higher adoption of TWT-2 and TWT-3, whereas the usage of TWT-1 was low (Figure 7).

## 4. Discussion

This study aimed to quantify the distribution of TAU and TWT adopted in neurorehabilitation programs of individuals with SCI with a specific focus on a selected Italian Spinal Cord Unit that includes PTs with at least three years of neurorehabilitation experience and who are trained and constantly updated in using technological devices. Results should be interpreted considering that they refer to a set of technologies specifically available in the setting under analysis and which clearly do not cover the full spectrum of solutions potentially available on the market or in other rehabilitation hospitals. Nonetheless, we think that our results can still be considered relevant for researchers and clinicians interested in the application of nonconventional solutions as add-ons to rehabilitation paths.

In this study, individuals with SCI were grouped according to two different approaches: a conventional method based on the AIS [3] level classification (AIS-based clustering) and a newly developed method that classifies individuals by taking multiple characteristics into account (MF clustering).

While the AIS-based classification is widely used, it does not fully capture the complexity of patients’ profiles. The AIS-based classification stems from its focus on neurological impairment, which does not account for the functional diversity within each AIS category. For example, individuals with the same AIS can exhibit different levels of independence in daily activities, influencing their rehabilitation needs. The MF clustering approach integrates functional factors, offering a more specific analysis of TWT adoption. This approach was conceived by considering that, besides neurological features, the range of functional improvements following SCI [38], as well as the rehabilitation goals and hospitalization duration, may vary even among individuals with the same AIS classification [60]. Indeed, even if individuals with SCI apparently can be considered comparable when classified according to the AIS level, they may actually achieve different ranges of functional recovery, also because they start from a different level of independence in the ADLs, especially after incomplete SCI [38]. The purpose of MF clustering was not to propose a new classification of neurological impairment that can replace the AIS-based approach but rather to provide better insights on technology adoption in the neurorehabilitation treatments of individuals with SCI by considering both neurological and functional recovery.

Nonetheless, the results of our analysis could be interesting from a future research point of view, since identifying homogeneous subgroups for clinical trials can help to more accurately determine whether a therapeutic intervention is actually effective [61]. The possibility in future studies to potentially develop a classification method able to group an intrinsically heterogeneous population in homogeneous subgroups, considering both neurological and functional information, can be advantageous for studies on SCI, where difficulties can arise due to the significant differences in the participants [60].

The analysis of the silhouette reveals a positive value for the MF clustering, suggesting a higher level of separation across the clusters in comparison to the negative score obtained for the four AIS-based clusters. These data are interesting since the AIS-based classification considers the neurological lesion in terms of completeness of the damage to the motor and/or sensory system (i.e., AIS level A, B, C, or D) [60]. Results indicate that MFc-1 includes almost all individuals with cervical or thoracic lesions classified as AIS A and B, whereas the individuals with lumbar complete lesions and with sensorimotor incomplete lesions were merged in the other two clusters: MFc-2 grouped thoracic and lumbar lesions, while MFc-3 included cervical ones. Therefore, in line with our initial hypothesis, the MF clustering was demonstrated to be capable of grouping the individuals based on neurological features and functional abilities. Individuals classified as AIS A and B, merged into the MFc-1, presented more severe functional injuries and less room for improvement, whereas individuals with sensorimotor incomplete SCI (AIS C and D) were grouped separately based on the lesion level. Indeed, considering that the lower the level of the injury, the greater the residual functional capacity, MFc-2 encloses less impaired individuals with respect to MFc-3. As reported above, this result does not claim to indicate that the proposed clustering approach can replace the AIS classification. Rather, it suggests that a more thorough analysis can be conducted on technology adoption in case functional capabilities and hospitalization length are taken into account besides strictly analyzing neurological state.

Despite these different groupings, results of our analysis showed that the amount of TWT is on average about 30% of the total therapies for all individuals, independently from the clustering approach. Although the overall percentage of technology adoption did not differ substantially among the clusters, some interesting differences may be highlighted by analyzing technology distribution on the basis of rehabilitation goals, that is, by comparing TWT-1, TWT-2, TWT-3, and TWT-4. Considering the AIS-based approach, for AISc-A and AISc-B, the most selected treatment goal was muscle tone control and modulation (TWT-1), while the other types of TWT were seldom adopted. Even for AISc-C and AISc-D, TWT-1 was the most frequent strategy since muscle tone modulation is pursued regardless of the AIS level. TWT-3 and mainly TWT-2 were proposed, respectively, to improve walking ability and balance control, particularly for individuals with thoracic or lumbar SCI. The main difference between AISc-C and AISc-D categories was that in the case of AISc-D, a higher level of BP and BPP adoption for balance rehabilitation and of TB Exo and TA FES for gait rehabilitation was observed. No statistical differences were noted across AIS-based clusters for TWT-4, suggesting a similar level of adoption of this category of technologies in the neurorehabilitation program.

The analysis of TWT adoption based on the MF clustering allows for a more insightful tuning of the neurorehabilitation (technology-aided or conventional) proposed to individuals with SCI by taking into account not only AIS level but also richer data. Indeed, AIS-based classification flattens important information on the functional status and on the independence in performing ADLs of each individual with SCI because it disregards the concrete possibility to have heterogeneous clinical and functional conditions within the same AIS level. In the case of MFc-1, there is almost exclusive use of TWT-1 technologies (about 94%), particularly the Ce device, and TWT-2 is the least utilized. Technologies dedicated to gait recovery are slightly more utilized than those for upper limbs. For MFc-2 and MFc-3, there is an almost similar use of technologies for muscle tone modulation (Ce devices). On the contrary, FES Ce was more adopted, and TWT-4 was never used for MFc-2. TWT-3 devices were more widely used in cluster MFc-3. All these differences were statistically significant, whereas no statistically significant difference among MF clusters was noted for TWT-2.

The technology used for muscle tone modulation, gait recovery, and upper limb function, was higher in individuals belonging to MFc-3 with respect to the ones of MFc-2 since they have a greater functional deficit. Moreover, individuals with cervical injury can benefit from a greater spectrum of technological solutions because of the possibility of taking advantage of tools for the upper limbs in addition to those for the lower limbs. Furthermore, individuals with cervical lesions remained hospitalized longer than individuals with thoracic or lumbar lesions belonging to AISc-B, AISc-C, and AISc-D. The lower the injury level, the shorter the average hospitalization duration in cases of sensorimotor incomplete lesions. This pattern is totally reversed only for AISc-A, for which longer stays are reported for lumbar lesions with respect to the cervical ones (Figure 1b). This may depend on the potential recovery and related functional goals planned to manage the discharge from the Spinal Cord Unit. For example, an individual with a complete cervical injury might have a relatively easy to reach rehabilitation goal, such as the autonomous management of a powered wheelchair. This goal might be achieved faster than the target of an individual with a lumbar complete lesion who aims to regain functional ambulation by using walking aids and braces. Moreover, for the MF clustering in the case of sensorimotor incomplete SCI (MFc-2 and MFc-3), individuals with cervical lesions (lower SCIM score) stay longer than thoraco-lumbar ones (higher SCIM score) into the Spinal Cord Unit. Overall, the individuals included in MFc-1 showed the lowest SCIM score at admission and remained hospitalized longer than the other two groups of individuals with incomplete injuries.

Analyzing TWT categories, the most frequently adopted device was the Ce, followed by FES Ce, independently from the clustering approach. The reason is probably due to the limited number of contraindications of these systems (almost all individuals are suitable for their adoption) and to their ease of application. These tools can be used without a direct intervention from PTs (i.e., by just relying on their supervision), thus also ensuring longer rehabilitation sessions. On the contrary, TWT-2, TWT-3, and TWT-4 were largely less utilized since they required the direct intervention from the PT. Indeed, not everyone was able to perform tasks more complex than those faced with TWT-1 devices, which simply required the patient to be sitting in the wheelchair or lying on the bed. Among the TWT-2 devices, the BP was used much more than the BPP, even though they both had quite similar functionalities when working in a standing position. This may be because BP can also be used in a seated position or even directly in the wheelchair in case of individuals unable to maintain standing. Hence, BP can be considered to have greater usability compared to the BPP. For technologies included in TWT-3, the TB Exo was more used than both the OG Exo and TA FES. Factors that may reduce the adoption of these two devices include the long time required for system setting and patient’s preparation, the nonnegligible spectrum of contraindications inherent in the type of device, and, consequently, the reduced number of individuals who could benefit from them. Moreover, for OG Exo, two PTs are simultaneously required to safely manage the user, whereas TA FES can be used in a reduced number of individuals since ambulation is not feasible for everybody and since its usage is quite specific for deficits to ankle dorsiflexor muscles. Regarding upper limb technologies, the MI BCI device was used more extensively than MT because it does not require motor engagement and thus allows all individuals, even those with severe upper limb impairments, to take advantage of it. For the sake of completeness, it is worth adding that actually, the validity of MI BCI for the recovery of upper limb function has been demonstrated for stroke survivors, and it is currently under investigation for individuals with SCI [55]. It is worth specifying that no difficulties or delays were encountered in the delivery of the abovementioned TWT, since the FSL clinical engineering department was constantly engaged in the maintenance of the devices to make them constantly working.

Although the main focus of this study was on quantifying technology adoption, the categorization of TAUs and related analysis provides interesting information. For both clustering methods, significant differences were found for TAU-1, TAU-2, and TAU-4 in the comparison among MF clusters and among AIS-based clusters. This indicates differences in the adoption of exercises focused on joint range of motion maintenance, muscle tone modulation and force control (TAU-1), transfers and trunk control (TAU-2), or gait ability (TAU-4). 

Indeed, conventional SCI rehabilitation is typically well addressed in many literature studies, but no clear data on the rehabilitation programs categorized by functional goals during hospitalization are available. The AIS-based clustering showed that for individuals grouped in AISc-A and AISc-B, a wide administration of TAU-1 and TAU-2 emerged. In this case, TAU-1 was proposed mainly to prevent the onset of secondary complications due to SCI [6], such as muscle shortenings or tone alterations, decreased range of motion, pressure sores, or neurogenic heterotopic ossification onset, and to only strengthen uninjured motor districts above the level of injury [62,63]. Moreover, TAU-2 was proposed to train transfers and trunk control with the purpose of promoting as much autonomy as possible. Besides TAU-1 and TAU-2, mainly TAU-3 was proposed to train individuals to reach and maintain standing position and to facilitate orthostatic hypotension control, promote bowel functionality, and improve bone mineralization by reducing the risk of severe osteoporosis and the occurrence of spontaneous fractures [64]. The amount of TAU-4 was very limited, since only a few individuals classified as AISc-A with low thoracic or lumbar injury can recover walking function by using braces, walking aids, and physical assistance, and only a small proportion of AISc-B individuals can recover walking function [65].

Even in the case of AISc-C and AISc-D, there was a prevalence of TAU-1, less than AISc-A and AISc-B clusters, which was mainly oriented towards strengthening the unimpaired districts and promoting the best motor recovery achievable below the lesion level. The second most frequently administered therapy was TAU-2, delivered to promote independence and to prepare the trunk control during most complex activities like standing or walking, followed by TAU-3. Individuals with SCI usually begin to experience upright standing position maintenance within parallel bars to ensure upper limb support in maintaining standing position, to allow the musculoskeletal system to accept the load progressively, and to promote proper bowel and bladder functions. TAU-4 was proposed more frequently to individuals in AISc-D than to those in AISc-C, since the former ones have the motor skills needed to walk again (with or without an aid, depending on the lesion) more suitable than all the other individuals [65].

Also for TAU, the analysis of MF clustering provides additional broader information. Overall, the highest level of TAU-1 adoption was reported for MFc-1, where almost no TAU-4 was noted. In MFc-3, there was a greater use of TAU-1 and TAU-4, at the expense of TAU-2, than in MFc-2. Hence, there is a greater focus on muscle tone and recruitment in addition to walking than on balance training, even if in MFc-3, there are only individuals with cervical SCI. No differences across MF clusters were observed for TAU-3, suggesting no specific differences in the balance of conventional rehabilitation.

The scientific literature on SCI neurorehabilitation states quite clearly that technologies should be considered as complementary aids to enhance the effectiveness of the conventional treatment [66]. Anyhow, the quantification of the adoption of technologies in clinical daily practice compared to conventional treatment has not been investigated in the field of SCI. Indeed, the scientific literature includes papers focusing on the analysis of device effectiveness or seldomly on the barriers, advantages, and disadvantages of their use, but no data on the actual technology adoption in daily clinical practice are available to the best of our knowledge. According to the results of the selected Italian Spinal Cord Unit, the amount of TWT is on average about 30% of the total therapies for all individuals with SCI, independently from the clustering approach. However, we cannot state if technologies can be considered underutilized or not because similar data on the actual adoption in daily rehabilitation is not easily traceable. Indeed, the only available data are mainly coming from mere indirect indications on market dimensions provided by manufacturers and market leaders related to their products [66]. On this basis, the literature generally suggests that the rate of technology adoption is quite limited due to a number of barriers [66], despite the fact that technology-aided neurorehabilitation presents several advantages, such as the possibility to perform intensive and task-oriented motor activities, to monitor and objectively quantify performance in real-time, and/or along time or to take advantage of continuous feedback, thus promoting motivation and engagement [67,68,69].

In spite of the big effort from developers to push new technologies in clinical environments, different factors have been identified as possible barriers contrasting their diffusion. The technological barriers may include the setup of the devices, the complexity of use, and the level of training necessary for PTs to master the technology [66]. Furthermore, the migration to technology-aided rehabilitation may be perceived as a de-personalization of treatment from the patient point of view, increasing the dislike and the impression to feel controlled by a machine. Users’ behaviors and motivations when using and interacting with rehabilitation devices may be considered key factors for the acceptance of individuals with SCI of the use of this technology. These factors include personal beliefs and perceptions of advantages and disadvantages of the use of a device [36]. Besides the patient’s point of view, the PTs may prefer long standing traditional rehabilitation methods or may be influenced by a limited knowledge of the available technologies and their potential functionalities. In fact, PTs should be constantly updated on innovations, which require learning new solutions and new characteristics and capabilities of the upgrades of the different devices [66]. Besides, the presence of expert personnel was perceived positively by individuals with SCI, primarily due to the sense of safety and trust they provided during the delivery of TWT [36]. This finding suggests that the involvement of skilled professionals is important in enhancing the overall experience and acceptance of technologies. From the organizational point of view, the introduction of technology-aided rehabilitation also requires dedicated training for the staff of PTs and, especially, an increase in the complexity in the management of therapeutic planning. Moreover, the adoption of new technologies often requires a reorganization of activities and redistribution of goals, interests, and responsibilities within the clinical organization, thus potentially increasing the resistance from the clinical staff. In the care of individuals with SCI, also medical doctors play a key role. The heterogeneity of technologies adopted within a neurorehabilitation framework requires not only the PTs but also, and maybe especially, the prescribing medical doctors to have a clear awareness of the potentialities of each technology, as well as of any exclusion criteria that prevent their use for specific individuals with SCI. Unfortunately, the scientific proofs of the benefits of all the available technology-aided training, or even the superiority over traditional solutions, are sometimes not clearly stated or controversial for the overall spectrum of SCI neurological features. Furthermore, there is a lack of well-defined guidelines to implement specific and effective protocols. To this aim, periodic team meetings are held in the FSL Spinal Cord Unit, in which the team, including medical doctors and PTs, evaluates the progress in using technological devices and makes decisions about continuing, modifying, or discarding ongoing therapies. Other possible limitations for many rehabilitation centers may be the overall costs for the purchase and maintenance of technologies or the questionable benefit-cost ratio. These uncertainties might support the preference for conventional therapy and possibly discourage investment in technologies.

Our study provided useful information to identify technologies adopted for the treatment of specific groups of individuals with SCI, at least according to what is commonly pursued during typical practice. Nonetheless, as anticipated, our results cannot be fully generalized since they are restricted only to a selected Italian Spinal Cord Unit with specific characteristics (number of PTs, years of experience in neurorehabilitation and technology device usage, internal scheduling procedures, etc.), and they do not cover a broad range of technologies available for clinical use. Moreover, the sample of individuals under analysis and the time window of observation can be considered relatively small. Hence, further studies are needed to deepen the topic of technology adoption. It would be interesting to reproduce this analysis in other SCI neurorehabilitation institutions to compare different Spinal Cord Unit in the same country, or even in different countries, considering the specificity of each environment. This would help understanding whether there are differences in technology adoption, which could be attributed to not only the complexity of the technology itself but rather also the difficulty in the usage or management for a specific pathology. Further investigations could be devoted to analyzing possible relationships between the amount of technology adoption and the actual effectiveness. The combination of data on the actual adoption of technologies in daily clinical practice coupled with effectiveness data could help for the selection of technologies to be acquired in a Spinal Cord Unit with related training courses for the professionals who will use them. Another possible interesting aspect to be investigated is the potential influence of TWT adoption on the hospitalization duration, given that the days of recovery are commonly used to measure the performance and efficiency of healthcare systems.

## 5. Conclusions

This study analyzed 104 individuals with SCI of a selected Italian Spinal Cord Unit in an observation time window of 29 months to assess the neurorehabilitation programs and quantify the distribution of conventional (TAU) and technology-aided (TWT) treatments, with a specific focus on categories of the available technological resources grouped based on different rehabilitation goals.

Results indicated as the main outcome an average technology adoption of about 30% of the overall rehabilitation sessions. Moreover, we confirmed the initial hypothesis that the use of the technologies changes among different clusters of individuals with homogeneous characteristics. In particular, we conducted a dual analysis by clustering individuals according to the conventional AIS level and to a new MF clustering approach that considers richer sources of information besides the AIS classification itself. MF clustering analysis was capable of grouping individuals based both on neurological features and functional abilities, grouping individuals with motor complete injuries (MFc-1), whereas individuals with sensorimotor incomplete SCI were gathered separately based on the lesion level (MFc2 and MFc3). As originally hypothesized, we found that MF clusters were more homogenous and less overlapped and that differences in the technology used were more pronounced than in AIS-based clustering. Nonetheless, we are not proposing a new classification model that can replace the AIS-based one, but suggesting that when investigating technology adoption in neurorehabilitation of individuals with SCI, it is convenient to also consider the functional capabilities and the hospitalization length. Across MF clusters, we found several statistically significant differences when comparing the adoption of TWTs or TAUs across clusters. In the case of motor-complete SCI, TWT for muscle tone control and modulation was mainly adopted, whereas the other types of TWT were seldom used. Even for incomplete SCI, the most frequent rehabilitation goal was muscle tone modulation, regardless of the AIS level, and technologies to improve walking ability and balance control were mainly used for individuals with thoracic or lumbar SCI. Analyzing TAU distribution, we found that the highest adoption of muscle tone modulation strategies was reported in the case of individuals with motor complete SCI, that is, in cases when almost no gait training was pursued. In the case of cervical motor incomplete SCI, compared to thoracic and lumbar incomplete SCI, there was a greater focus on increasing muscle recruitment in addition to walking than on balance training.

## Figures and Tables

**Table 1:** Available technologies at FSL Spinal Cord Unit classified into Treatment With Technologies (TWTs) categories.

Treatment with Technologies (TWTs) Category	Device	Device Acronym
TWT-1	Cycloergometer (Ce)	Ce
	Functional Electrical Stimulation (FES)-aided Cycloergometer (Ce)	FES Ce
TWT-2	Balance Plate (BP)	BP
	Balance Perturbation Plate (BPP)	BPP
TWT-3	Tibialis Anterior (TA) Functional Electrical Stimulation (FES)	TA FES
	Lower limb Treadmill-Based exoskeleton (TB exo)	TB exo
	Lower limb Overground exoskeleton (OG exo)	OG exo
TWT-4	Upper limb Motor Imagery (MI) Brain Computer Interface (BCI)	MI BCI
	Upper limb Game-based Motor Trainer (MT)	MT

## Data Availability

Data are available upon reasonable request to the corresponding author.
